# Genetic Contribution to Non-alcoholic Fatty Liver Disease and Prognostic Implications

**DOI:** 10.1007/s11892-021-01377-5

**Published:** 2021-02-05

**Authors:** Katherine Martin, Anas Hatab, Varinder S. Athwal, Elliot Jokl, Karen Piper Hanley

**Affiliations:** 1 Wellcome Centre for Cell-Matrix Research, Faculty of Biology, Medicine & Health, Manchester Academic Health Science Centre, University of Manchester, Oxford Road M13 9PT, Manchester, UK; 2 Division of Diabetes, Endocrinology and Gastroenterology, Faculty of Biology, Medicine & Health, Manchester Academic Health Science Centre, University of Manchester, Oxford Road, Manchester, UK; 3 Manchester University NHS Foundation Trust, Oxford Road M13 9PT, Manchester, UK

**Keywords:** Non-alcoholic fatty liver disease, Genetic, Risk stratification

## Abstract

**Purpose of Review:**

Non-alcoholic fatty liver disease (NAFLD) is a major and increasing health burden, with the potential to overwhelm hepatology services. However, only a minority of patients develop advanced liver disease. The challenge is early identification of patients at risk of progression. This review aims to summarize current knowledge on the genetic predisposition to NAFLD, and its implications for prognostication and risk stratification.

**Recent Findings:**

*PNPLA3*-I148M is the most robustly associated genetic variant with NAFLD. Recently, variants in *TM6SF2*, *MBOAT7*, *GCKR* and *HSD17B13* have also been implicated. NAFLD is a complex disease, and any one genetic variant alone is insufficient for risk stratification, but combining multiple genetic variants with other parameters is a promising strategy.

**Summary:**

It is anticipated that, in the near future, analysis of data from large-scale prospective cohorts will reveal NAFLD subtypes and enable the development of prognostic models. This will facilitate risk stratification of patients, enabling optimisation of resources to effectively manage the NAFLD epidemic.

## Introduction

Non-alcoholic fatty liver disease (NAFLD) is now the most common liver disease in Europe and the United States and affects an estimated 25% of the global population [1], increasing to 55% in those with type 2 diabetes [2]. Although the majority of patients do not develop clinically significant liver disease, the high prevalence means that NAFLD is a heavy healthcare burden [3, 4]. It is already the second leading cause of liver transplantation in the United States [5], with mortality expected to more than double by 2030 [6].

**Fig. 1:**
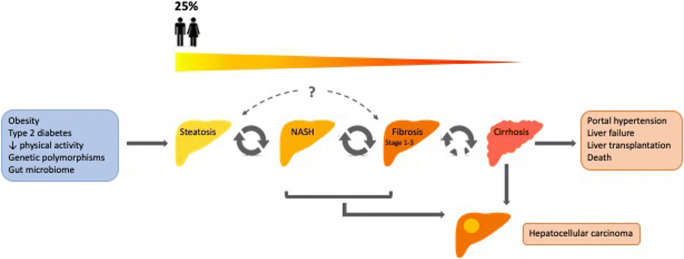
NAFLD encomapasses as a spectrum of disease, ranging from steatosis through NASH, to fibrosis and cirrhosis. 25% of the population are estimated to have NAFLD [1]. Only a minority will develop advanced disease, but large-scale long-term studies are needed to better understand the risk of progression. Factors promoting the development and progression of NAFLD include obesity, type 2 diabetes, reduced physical activity, genetic variants and alterations of the gut microbiome. Conversely, weight reduction and exercise can result in disease regression. Cirrhosis is associated with risks of portal hypertension, liver failure, liver transplantation and death. HCC typically arises on a background of cirrhosis, but in NAFLD it can also occur pre-cirrhosis. NAFLD, non-alcoholic fatty liver diseases; NASH, non-alcoholic steatohepatitis; HCC, hepatocellular carcinoma

NAFLD is characterized by steatosis (>5% hepatocytes) in the absence of significant alcohol intake or secondary causes (e.g. steatotic drugs); and encompasses a spectrum of disease ranging from simple steatosis through steatohepatitis (nonalcoholic steatohepatitis, NASH: steatosis plus inflammation and hepatocyte ballooning) to fibrosis, cirrhosis and hepatocellular carcinoma (HCC). There is considerable heterogeneity in disease phenotype, and the natural history and risk of progression to clinically significant liver disease are uncertain. Patients may have stable disease, progress slowly or rapidly, or even regress [7]; some will develop HCC [8] (Fig. 1).

Understanding which patients are at risk of liver-related morbidity and mortality is essential. Firstly, risk stratification of patients to appropriate follow up is necessary to manage the burden of disease. Secondly, subtyping patients based on risk of progressive disease will facilitate baseline parameter standardization in clinical trials, enabling identification of effective treatments [9]. Thirdly, understanding individualized risk will help clinicians select those patients most likely to benefit from new drug therapies, as they become available.

## NAFLD Pathogenesis

NAFLD is a complex disease resulting from interplay of genetic, environmental, metabolic and microbial factors. The rising prevalence of NAFLD is linked to the increasing trends in obesity and type 2 diabetes, resulting from changes in dietary patterns and sedentary lifestyles [10]. NAFLD is closely linked to the metabolic syndrome, a cluster of conditions including central obesity, hypertension, dyslipidaemia and hyperglycaemia [11]. The major source of hepatic lipid is through lipolysis of adipose tissue [12]. Basal lipolysis is enhanced in obesity, leading to increased delivery of free fatty acids to the liver and skeletal muscle [13]. In turn, this may promote insulin resistance [13, 14]. Secondary sources of hepatic lipid are de novo lipogenesis from excess dietary sugars, and dietary fat [12].

Within the liver, free fatty acids are metabolized by mitochondrial beta-oxidation or incorporated into triglycerides, for export as very low density lipoprotein (VLDL) or sequestering into lipid droplets [15]. When the liver’s capacity to metabolize free fatty acids is exceeded, harmful lipid species may be formed, which can activate lipotoxic pathways leading to hepatocellular injury and driving progression towards NASH [15–18].

Fibrosis, or scarring, results from a dysregulated wound healing response to repeated hepatocellular injury. Inflammatory mediators activate hepatic stellate cells to myofibroblasts, which migrate into the liver parenchyma and secrete the collagen-rich extracellular matrix that characterizes fibrosis [19]. At the same time, mechanisms for fibrolysis are inhibited, leading to a net increase in scarring [19]. Ultimately, this leads to cirrhosis, characterized by thick, fibrous septae and architectural distortion, which predisposes to the complications of portal hypertension, organ failure and HCC. With removal of the injurious agent, fibrolysis may be favoured, and even cirrhosis can regress [20].

## Disease Progression

Previously, simple steatosis, without evidence of NASH, was thought to be a benign condition, with no risk of progression to clinically significant liver disease. However, there have been several studies and systemic analyses evaluating disease progression with paired liver biopsies, which challenge this assumption.

Staging of NAFLD fibrosis is usually assessed using the NASH Clinical Research Network histological scoring system [21]. This ranges from no fibrosis (stage 0), through mild (stage 1), moderate (stage 2), and advanced fibrosis (stage 3; bridging fibrosis, spanning portal-to-portal or portal-to-central spaces), to cirrhosis (stage 4).

In a large meta-analysis, Singh et al. demonstrated that 39.1% of those with steatosis but no evidence of NASH developed progressive fibrosis, with an average progression rate of one fibrosis stage every 14 years [7]. On average, patients with NASH progressed twice as fast. Importantly, they identified a subgroup of ‘rapid progressors’, with one in five progressors advancing from no fibrosis at baseline to advanced fibrosis or cirrhosis over a mean 5.9 years [7]. More recent studies have also demonstrated progression to advanced fibrosis or cirrhosis in around 20% of patients with baseline steatosis without evidence of NASH, over an average follow up of 4 to 8 years [22, 23]. Overall, 34% of patients have progressive disease, 43% have stable disease and 22% will regress [7].

However, many of these studies are retrospective, performed in tertiary centres, and limited by inherent selection bias in patients undergoing repeated liver biopsy. Therefore, it may be difficult to extrapolate these findings to the general population. To address this, Loomba et al. studied a large, real-world cohort of patients in the United States, and identified that 39% of those with an initial diagnosis of NAFLD progressed to advanced liver disease (cirrhosis, liver transplant or HCC) over an 8 year study period [24]. It has been argued that this may overestimate the risk of progression to advanced liver disease, due to the low prevalence of NAFLD (5.7%; perhaps due to underdiagnosis) and older age (≥65 years; favouring fibrosis progression) of the study cohort [25]. Other population-based studies are currently underway to ascertain the prevalence of chronic liver disease and identify population subgroups at highest risk of progressive fibrosis [25].

## Assessing the Disease Stage

Liver biopsy is the gold standard for diagnosing NASH and staging fibrosis. However, it is limited by sampling error (liver pathology can be heterogeneous, and biopsy only samples approximately 1/50,000th of the liver), and inter-observer variability [26]. It is also associated with cost, inconvenience, and risk to the patient, including very rarely, a risk of death [26]. Moreover, it has been suggested that rather than following a linear course, NASH activity may fluctuate over time, and therefore, a singular liver biopsy may not accurately reflect disease severity [27].

For these reasons, there is a major research focus to identify and validate non-invasive fibrosis biomarkers. Several have been developed, including specific blood biomarkers (e.g. Enhanced Liver Fibrosis test); combined scores based on clinical data and standard laboratory investigations (e.g. NAFLD Fibrosis Score); and imaging modalities (e.g. transient elastography, Fibroscan). Whilst currently available biomarkers are useful for ruling out advanced fibrosis, they perform less well at diagnosing cirrhosis and discriminating between fibrosis stages [28]. This means that many patients still require a liver biopsy for an accurate diagnosis.

Currently, the British Society for Gastroenterology (BSG) and European Association for Study of the Liver - Asociacion Latinoamericana para el Estudio del Higado (EASL-ALEH) guidelines advocate using non-invasive biomarkers to assess advanced fibrosis in NAFLD [29, 30]. Patients are then dichotomised into those at low or high risk of advanced fibrosis. Patients at low risk are managed in primary care, with repeat assessment of fibrosis risk every 2 to 5 years; patients at high risk are referred to hepatology services for further assessment [29].

Liver fibrosis is the strongest predictor of liver-related and all-cause mortality in NAFLD [31–33]. This underpins the current diagnostic pathway, with only patients at high risk of advanced fibrosis referred to hepatology services. Liver disease is typically asymptomatic until complications develop. The majority of patients with cirrhosis are first diagnosed during a hospital admission with decompensated disease [34]. Identifying asymptomatic patients with cirrhosis would allow opportunity for monitoring and treatment for complications, including surveillance for varices and HCC. However, the current pathway is suboptimal. Firstly, patients with low risk of advanced fibrosis who will never go on to develop clinically significant liver disease may be needlessly followed up, creating unnecessary workload and potentially fuelling health related anxiety. Secondly, patients at high risk of fibrosis progression may miss the opportunity for intensive management to minimize their risk of developing cirrhosis. To address this, we need methods of identifying those patients with early disease who are destined to progress.

Although HCC typically develops on a background of cirrhosis, in NAFLD it can occur in non-cirrhotics. Up to 49% of NAFLD-related HCC occurs in patients without background cirrhosis [8]. NAFLD-related HCC presents late, with a more advanced tumour stage, and a poorer prognosis [35]. Currently, there is no recommendation for HCC surveillance in patients with NAFLD without cirrhosis [36]. Understanding which patients are most at risk, may allow for earlier diagnosis and improvement in outcomes.

## Predicting Risk: Key Genes

### PNPLA3

Genetic risk for NAFLD susceptibility is suggested by ethnic variability [37], increased risk with a parental history [38], and twin studies [39, 40]. Notably, a twin study in the United States has shown that the heritability of hepatic steatosis and fibrosis is around 50% [40]. Genome wide association studies (GWAS) look for links between common genetic variants (single nucleotide polymorphisms, SNPs) and disease phenotypes. Over the last decade, a number of GWAS have revealed genetic variants associated with NAFLD. The first, by Romeo et al., identified that a SNP (rs738409) in the gene encoding patin-like phospholipase domain-containing protein 3 (PNPLA3) was strongly associated with hepatic steatosis and inflammation (assessed by liver transanimases) [41^••^]. The rs738409 variant is a cytosine to guanine substitution, which results in a switch from isoleucine to methionine at residue 148 (I148M). *PNPLA3* is predominantly expressed in the liver and retina [42]. In vitro, PNPLA3 catalyses the hydrolysis of triglycerides [43]. Its catalytic activity is disrupted by the I148M mutation. However, *Pnpla3*3 knockout mice do not develop hepatic steatosis [44]. Therefore, PNPLA3-I148-mediated steatosis is not thought to result from a simple loss-of-function. Instead, PNPLA3-I148M resists degradation and accumulates on lipid droplets [45], where it is thought to sequester the lipase co-factor comparative gene identification-58 (CGI-58), thereby indirectly inhibiting other lipases [46, 47].

PNPLA3 is more highly expressed in hepatic stellate cells in the liver, where it is also involved in the metabolism of intracellular lipid droplets [42]. Quiescent hepatic stellate cells store retinol in the form of retinol palmitate. PNPLA3 has been shown to hydrolyse retinol palmitate and promote the release of retinol from hepatic stellate cells [42]. Hepatic stellate cells are activated to myofibroblasts in response to liver injury. This activation process is associated with loss of their intracellular lipid droplets [48]. The *PNPLA3*-I148M variant is associated with a reduction in lipid droplet metabolism [42], and a more inflammatory and fibrogenic phenotype in hepatic stellate cells in vitro [49].

*PNPLA3*-I148M has subsequently been associated with all aspects of NAFLD, including age at diagnosis [50]; hepatic steatosis [51]; disease severity [51]; fibrosis stage [51]; and HCC [52]. Most recently, in the largest GWAS on histologically characterized NAFLD, *PNPLA3*-I148M was confirmed to be associated with the full spectrum of disease [53^••^].

Interestingly, carriage of the *PNPLA3*-I148M variant confers a poorer prognosis in other liver diseases including alcohol-related liver disease [54], and autoimmune hepatitis [55]. Alcohol excess causes hepatic steatosis, and therefore, in alcohol-related liver disease, *PNPLA3*-I148M may increase risk of progression through a shared mechanism with NAFLD. However, in autoimmune hepatitis the effect of PNPLA3-I148M was seemingly unrelated to hepatic steatosis, suggesting alternative mechanisms of action [55].

Genotyping for the *PNPLA3*-I148M variant is not recommended as a singular test for risk stratification of NAFLD or HCC [36, 50, 52]. Indeed, its effects are modulated by interactions with environmental factors and other gene variants. For example, its effect is potentiated by adiposity: Stender et al. showed that homozygosity for *PNPLA3*-I148M variant was associated with hepatic steatosis in 18% of lean individuals compared with 84% in the very obese [56]. *PNPLA3*-I148M is also modified by interaction with other genetic polymorphisms. Donati et al. discovered that an additional polymorphism in the *PNPLA3* gene*, rs2294918 G > A* encoding the E434K protein variant, ameliorated the effect of PNPLA3-I134M on development of NASH by reducing its expression [57].

### TM6SF2

A polymorphism (rs58542926 A > G) in the transmembrane 6 superfamily member 2 (*TM6SF2*) gene is associated with hepatic steatosis and progressive fibrosis [53, 58–60]. The genetic variant results in a substitution of lysine for glutamate at residue 167 [59]. The function of the protein was unknown. However, recent studies in mice have revealed that loss of *Tm6sf22* is associated with reduced hepatic lipid secretion via VLDL, with excess lipid accumulating in hepatocellular droplets [61]. When fed a normal diet, the *Tm6sf2* knockout mice developed hepatic steatosis, elevated liver enzymes and hypocholesterolaemia, recapitulating the human phenotype [61]. Interestingly, loss of *Tm6sf22* was associated with a marked reduction in expression of PNPLA3 [61]. Notably, the *TM6SF2* variant is associated with lower levels of circulating total cholesterol, LDL-cholesterol and triglycerides, and is protective against cardiovascular disease [60, 62]. Therefore, targeting *TM6SF2* therapeutically may not be viable in NAFLD.

### MBOAT7

The rs641738 C > T polymorphism in the membrane bound O-acyltransferase domain containing 7 (*MBOAT7*) gene was initially identified as a genetic modifier of risk for alcohol-related cirrhosis [54]. Subsequently, it was shown to be associated with hepatic steatosis, and severity of NAFLD-related necroinflammation and fibrosis [63]. More recently, it has been associated with NAFLD-related HCC, particularly in non-cirrhotics [64]. However, others have not found evidence of an association between *MBOAT7* and NAFLD [53, 65].

The *MBOAT7* gene encodes the enzyme lysophosphatidylinositol acyltransferase 1 (LPIAT1), which catalyses the incorporation of arachidonic acid into phosphatidylinositol [66]. The rs641738 variant is associated with a reduction in both mRNA and protein MBOAT7/LPIAT1 levels [63], but the mechanisms linking this to the development of NAFLD are unclear. Recently, Tanaka et al. have discovered a novel pathway. They demonstrated that loss of MBOAT7/LPIAT1 is associated with increased triglyceride synthesis and accumulation in hepatocytes, secondary to increased phosphatidylinositol turnover resulting in increased production of diacylglycerol, a substrate for triglyceride synthesis [67]. In addition, loss of *MBOAT7*/*LPIAT1* in hepatic stellate cells was associated with a more fibrogenic phenotype [67].

On the contrary, Thangapandi et al. demonstrated that hepatocyte-specific *Mboat7* deletion in mice is associated with increased hepatocyte cholesteryl esters but not triglycerides [68]. In addition, they discovered that hepatocyte-specific *Mboat7*deficient mice develop hepatic fibrosis in the absence of inflammation when fed a NAFLD-inducing diet. Similarly, in patients with a BMI ≤ 35, carriage of the rs641738 polymorphism was associated with hepatic fibrosis independent of inflammation [68].

### GCKR

In the liver, glucokinase regulatory protein (GKRP) regulates the activity of glucokinase, depending on the glycaemic levels. In low glucose conditions, GKRP binds to, and sequesters, glucokinase in the hepatocyte nucleus [69]. Whereas in high glucose conditions, GKRP is released from glucokinase, allowing its translocation from the nucleus to the cytoplasm [69]. Cytosolic glucokinase activates glucose storage pathways including glycogen synthesis and de novo lipogenesis [70]. Counterintuitively, overexpressing GKRP in diabetic mice results in decreased fasting blood glucose levels and improved insulin sensitivity [69]. It is proposed that GKRP may also act to stabilize glucokinase protein, thereby leading to increased levels and activity [69].

The SNP rs1260326 in the *GCKR* gene encodes a C to T substitution, which results in a switch from proline to leucine at residue 446 (P446L) [71]. Functionally, this attenuates the capacity of GCKR to inhibit glucokinase [70]. Initially, the *GCKR*-P446L variant was linked to increased triglyceride levels, but reduced fasting glucose, insulin resistance and type 2 diabetes risk [72]. Subsequently, it has been associated with hepatic steatosis [53, 71]. A further SNP in the *GCKR* gene (rs780094) has also been shown to associate with NAFLD and fibrosis severity [73–75]. However, from studies in type 2 diabetes, these two SNPs are in strong linkage disequilibrium [76, 77].

### HSD17B13

Hydroxysteroid 17-beta dehydrogenase 13 (HSD17B13) is a liver-specific, lipid droplet associated protein [78, 79], with retinol dehydrogenase activity in vitro [53, 80]. It is upregulated in human liver tissue in NAFLD, and its overexpression results in the development of hepatic steatosis in mice [79]. Several studies have now reported that loss-of-function variants in *HSD17B13* are protective against the development of NASH and NAFLD cirrhosis [53, 80, 81]. However, the mechanisms underlying this remain to be elucidated.

## Polygenic Risk Scores

NAFLD is a complex disease, therefore, it is logical that combing genetic variants into a risk score will improve prognostic accuracy over a singular genetic variant. Recently, Gellert-Kristensen et al. demonstrated that a genetic risk score, combining the three genetic variants in *PNPLA3*, *TM6SF2* and *HSD17B13*, was associated with risk of cirrhosis and HCC in fatty liver disease (both NAFLD and alcohol-related) in the general population [82]. The score ranged from 0 to 6 depending on the number of risk alleles; a score of 5 or 6 was associated with a 12-fold increased risk of cirrhosis and a 29-fold increased risk of HCC [82]. Although, of note, only 0.5% of the study population scored a 5 or 6 [82].

In their editorial, Pfeiffer et al. demonstrated that, despite its strong association, Gellert-Kristensen et al.’s genetic risk score has limited usefulness as a singular test for risk stratification, due to its low positive predictive value (0.003 for cirrhosis and 0.0008 for HCC for score ≥ 4 in the UK population) [83]. However, it is anticipated that risk prediction scores will be used in confirmed NAFLD (rather than the general population), to stratify patients to appropriate follow up, and target individuals for novel therapeutics and enhanced surveillance. It is likely that genetic risk variants will need to be combined with other variables, such as clinical parameters, to improve score performance [83]. Similarly a genetic risk score including variants in *PNPLA3*, *TM6SF2*, *HSD17B13* and *GCKR* found a significant association with steatosis, steatohepatitis and fibrosis in a large cohort of patients with histologically characterized NAFLD [53^••^]. Gellert-Kristensen et al. also evaluated inclusion of the *GCKR* variant, but found that it did not improve score performance [82].

## Conclusion

Our knowledge of the aetiological drivers and pathophysiology of NAFLD has increased greatly in recent years. However, translating this to clinical practice remains a challenge. The majority of patients with NAFLD will not develop advanced liver disease. Therefore, identifying at-risk individuals to target for therapeutics and enhanced surveillance is critical. For this, the most accurate risk prediction scores will combine genetics with clinical variables and other biomarkers reflecting the underlying pathological mechanisms. However, accuracy will need to be weighed against cost, ease and acceptability for large-scale implementation.

In the near future, it is likely that artificial intelligence-based strategies to interrogate large multimodal datasets will uncover disease subtypes and enable the development of prognostic models. This will facilitate risk stratification of patients, optimisation of resources, and individualized treatment. This should, of course, be combined with wider, governmental strategies to address the root causes of the epidemic (e.g. societal and environmental factors promoting obesity) [4, 84].
